# Associations between subspecialty fellowship interest and knowledge of internal medicine: A hypothesis-generating study of internal medicine residents

**DOI:** 10.1186/1472-6920-11-5

**Published:** 2011-01-31

**Authors:** Uchenna R Ofoma, Erik E Lehman, Paul Haidet, Andrew C Yacht

**Affiliations:** 1 Department of Internal Medicine, Penn State College of Medicine, 500 University Drive, Hershey, PA 17033, USA; 2 Department of Public Health Sciences, Penn State College of Medicine, 500 University Drive, Hershey, PA 17033, USA; 3 Department of Internal Medicine, Maimonides Medical Centre, 4802 Tenth Avenue, Brooklyn, NY 11219, USA

## Abstract

**Background:**

Little is known about whether and how medical knowledge relates to interest in subspecialty fellowship training. The purpose of this study was to examine the relationships between residents' interest in subspecialty fellowship training and their knowledge of internal medicine (IM).

**Methods:**

A questionnaire was emailed to 48 categorical postgraduate-year (PGY) two and three residents at a New York university-affiliated IM residency program in 2007 using the Survey Monkey online survey instrument. Overall and content area-specific percentile scores from the IM in-training examination (IM-ITE) for the same year was used to determine objective knowledge.

**Results:**

Forty-five of 48 residents (response rate was 93.8%) completed the survey. Twenty-two (49%) were PG2 residents and 23(51%) were PGY3 residents. Sixty percent of respondents were male. Six (13%) residents were graduates of U.S. medical schools. Eight (18%) reported formal clinical training prior to starting internal medicine residency in the U.S. Of this latter group, 6 (75%) had training in IM and 6 (75) % reported a training length of 3 years or less. Thirty-seven of 45 (82%) residents had a subspecialty fellowship interest. Residents with a fellowship interest had a greater mean overall objective knowledge percentile score (56.44 vs. 31.67; p = 0.04) as well as greater mean percentile scores in all content areas of IM. The adjusted mean difference was statistically significant (p < 0.02) across three content areas.

**Conclusions:**

More than half of surveyed residents indicated interest in pursuing a subspecialty fellowship. Fellowship interest appears positively associated with general medical knowledge in this study population. Further work is needed to explore motivation and study patterns among internal medicine residents.

## Background

The 3-year internal medicine residency training format used by programs in a number of countries aims to produce quality internists with broad general knowledge. The scope of internal medicine has expanded tremendously over the past few decades. Amid this context, there is growing consensus that residency education in internal medicine urgently needs to be redesigned to accommodate new regulations (e.g., duty hour restrictions), and to better prepare internists for the rapidly evolving system of health care delivery and the expanding body of medical knowledge, prompting many professional organizations including the Society of General Internal Medicine (SGIM), the American College of Physicians (ACP) and the Association of Program Directors in Internal Medicine (APDIM) to write position papers on the issue [1-10].

Over the last decade, there has been an attempt at reform by way of a shift in focus to outcomes-based education in residency training [11]. However, the overall adequacy of internal medicine education is a composite of structure, process and outcomes. While the burden of designing the structures and processes to achieve the desired outcomes still rests with medical educators [12], the Accreditation Council for Graduate Medical Education (ACGME) has provided a framework for the desired outcomes by defining core competencies required of physicians.

In addition to the expanded scope, other factors also fuel current concerns about graduate training in internal medicine. Among these are the quality of resident education and the failure of the current system to address needs related to the trainee's ultimate career plans [9]. Authorities like the SGIM have therefore advocated that the current 3-year internal medicine training format be lengthened to 4 years [5], while subspecialists and subspecialty groups like the Cardiology Working Group 8 have proposed shortening the length of residency so that residents can quickly advance to subspecialty training [13-15]. Amid such debate, the American College of Physicians, which is closely aligned with accreditation bodies for internal medicine residency and fellowship programs in the United States is in favour of maintaining the status quo 3-year model, with a proviso to provide "core" training focused on general internal medicine in the first 2 years and customized training tailored to ultimate career goals in the last year [9].

Subspecialty fellowship applications remain increasingly competitive and internal medicine (IM) residents are often compelled early in training to devote much time and energy to their subspecialty of interest by way of study and research. How much of the "core" medical curriculum do they learn in this process? Relatively little is known about how general medical knowledge is associated with interest in subspecialty fellowship training among IM residents. The purpose of this study, therefore, was to determine the relationships between expressed subspecialty fellowship interest and knowledge of internal medicine. We hypothesized that residents with fellowship interest would have lesser knowledge of IM than their counterparts without fellowship interest.

## Methods

The study was designed as a prospective questionnaire-based cross-sectional survey. We distributed a questionnaire to all categorical internal medicine residents in a New York, university-affiliated IM residency training program in December 2007. We prepared the electronic questionnaire and database of potential respondent email addresses using the Survey Monkey online survey instrument and emailed a link to the online questionnaire to each potential participant. Potential respondents received one to two personal reminder calls if they did not respond to the initial survey within a week. If residents expressed a desire not to participate in the survey, they did not receive any additional reminder calls. Residents were not coerced in any way to complete the survey and received no compensation or inducements. The survey instrument consisted of 7 sections and 19 questions. The average time reported by residents to complete the survey was ten minutes. The 7 sections of the survey collected basic demographic data (age, gender, year of training, country of medical school, medical school graduation year and history of prior formal clinical training); information pertaining to fellowship choices, resident study patterns and post-residency practice plans. (See additional file 1)

The survey used a skip logic pattern, allowing residents to skip certain portions based on responses to preceding questions. The primary independent variable was interest in a subspecialty fellowship, defined as current or previous participation in a previous fellowship application process (irrespective of whether the resident was accepted to the fellowship). Non-interest in fellowship was defined by not having participated in a fellowship application process, irrespective of future intent. The primary outcome of interest was actual medical knowledge as measured in the yearly Internal Medicine In-Training Examination (IM-ITE) [16]. The IM-ITE is an educational program sponsored jointly by the American College of Physicians (ACP), the Association of Professors of Medicine (APM), and the Association of Program Directors in Internal Medicine (APDIM). The examination emphasizes a range of content areas considered important during the training of a general internist, including cardiology, gastroenterology, endocrinology, pulmonology/critical care medicine, rheumatology, nephrology, hematology/oncology, infectious disease, neurology, geriatric medicine, and general internal medicine (including, but not limited to, dermatology, ophthalmology, preventive medicine, psychiatry, geriatrics, women's health, nutrition, medical ethics, and biostatistics). The reliability and validity of the IM-ITE has been previously reported [17]. This 1-day examination was administered at the institution of study in October of 2007. Percentile scores from the IM In-Training Examination (IM-ITE) for the same year were utilized to objectively determine overall medical knowledge and knowledge of the various content areas of internal medicine, including general internal medicine. We analyzed the data with residents assigned to specialty fellowship interest (FI+) or not (FI-) regardless of residents' success in securing fellowship positions (i.e. an "educational intention" analysis). The Institutional review board approved the protocol and residents gave individual written consent for the release of IM-ITE scores. IM-ITE scores were de-identified and matched to the appropriate questionnaire by an independent party prior to data analysis, thereby ensuring confidentiality.

All analyses were conducted using SAS statistical software, version 9.2 (SAS Institute, Cary, NC). Descriptive statistics were computed for each item in the survey using means for continuous variables and percentages for categorical variables. A Pearson exact chi-square test was used to analyze the association between fellowship interest and the demographic variables and to analyze factors influencing study pattern. Mean objective knowledge percentile scores were compared between those with fellowship interest and those without fellowship interest using a Two-sample T- test. Non-parametric analyses comparing these same variables yielded similar results to parametric tests, despite the relatively small sample size. Two-sample t-tests are therefore reported. Analysis of variance (ANOVA) was utilized to test for confounding relationships between demographic variables and the objective knowledge percentile scores. Logistic regression was used to examine the association of the demographic variables with fellowship interest. An Analysis of Covariance (ANCOVA) was applied to the comparison of mean objective knowledge percentile scores between the two groups to adjust for demographic variables that had a statistically or plausible significant relationship with the scores. Being a hypothesis-generating pilot study, adjustments for multiple comparisons were not performed.

## Results

The survey was distributed to all 48 categorical internal medicine residents (24 in each of the second and third postgraduate years of training). Response rate was 45 of 48 (93.8%). Table 1 summarizes the demographics of respondents.

**Table 1:** Demographics of Survey Respondents

	Total	FI+	FI-	P-value*
**Variable**	**N (%)**	**N (%)**	**N (%)**	

**Total**	45 (100)	37 (82)	8 (18)	

**PGY**				

2	22 (49)	15(68)	7(32)	0.02

3	23 (51)	22(96)	1(4)	

**Age**				

29 or less	24 (53)	21 (88)	3 (12.5)	0.04

30-34	16 (36)	14 (88)	2 (12.5)	

35-39	5 (11)	2 (40)	3 (60)	

40 or older	0 (0)	0 (0)	0 (0)	

**Gender**				

Male	27 (60)	24 (89)	3 (11)	0.24

Female	18 (40)	13 (72)	5 (28)	

**CMS**				

US Grad	6 (13)	5 (83)	1 (17)	1.00

IMG	39 (87)	32 (82)	7 (18)	

**Years Since Grad**				

2 or less	18 (40)	13 (72)	5 (28)	0.16

3-5	19 (42)	18 (95)	1 (5)	

6 or more	8 (18)	6 (75)	2 (25)	

**Previous Clinical Training (PCT)**				

Yes	8 (18)	7 (88)	1 (12)	1.00

No	37 (82)	30 (81)	7 (19)	

Length of PCT				

0-1	3 (38)	3 (100)	0 (0)	1.00

2-3	3 (38)	2 (67)	1 (33)	

≥4	2 (24)	2 (100)	0 (0)	

**Specialty of Previous Training**				

Internal Medicine	6 (75)	5 (83.3)	1 (17)	1.00

Other	2 (25)	2 (100)	0 (0)	

*Pearson Exact Chi-Square Test

Of the 45 respondents, 27 (60%) were male and 18(40%) female. Respondents were equally divided between the two postgraduate years of residency training (49% & 51%). Six respondents (13%) were graduates of U.S. medical schools. Eight (18%) respondents reported prior formal clinical training prior to starting internal medicine residency in the United States. Of this group, 6 (75%) had prior training in Internal Medicine. 75% of respondents with prior formal clinical training reported a training length of 3 years or less. 60% of all respondents reported having at least three years elapse since graduation from medical school and prior to starting residency training. Thirty-seven of the 45 respondents (82%) indicated interest in subspecialty fellowship training. Of these, 60.5% committed to their fellowship choice in the first two years of current residency training, while 30% committed prior to residency. 72% of respondents had at least 2 months of clinical experience in their field of fellowship interest. The three subspecialties with the highest interest were Cardiology (28%), Pulmonary and Critical Care (19%), and Hematology and Oncology (12%), while 75% of IM residents with fellowship interest spent at least 2-3 months rotating through their sub-specialty interest areas (Table 2).

**Table 2:** Other characteristics of residents with fellowship interest

Variable	N (%)
**Rotation Experience in field of fellowship choice**	

0 months	1 (3)

Less than 1 month	3 (8)

1 month	5 (14)

2-3 months	19 (53)

4 or more months	8 (22)

**Fellowship specialty**	

Cardiology	10 (28)

Hematology and Oncology	5 (14)

Infectious Diseases	2 (6)

Nephrology	3 (8)

Gastroenterology	2 (6)

Rheumatology	1 (3)

Pulmonary Medicine with Critical Care	7 (19)

Critical Care with Other Specialty	3 (8)

General Internal Medicine	3 (8)

With the exception of age (p = 0.04) and post-graduate year of training (PGY: p = 0.02), groups of internal medicine residents with and without fellowship interest did not differ significantly: gender (p = 0.24), years since graduation (p = 0.16), country of medical school, prior formal clinical training as well as in specialty of prior formal clinical training (p = 1.0).

Internal medicine residents with fellowship interest had greater unadjusted mean overall percentile scores (p < 0.01) as well as greater unadjusted mean scores in all content areas of internal medicine. The unadjusted mean difference was statistically significant (p < 0.05) across all content areas with the exception of General Internal Medicine, Gastroenterology and Rheumatology. (Table 3)

**Table 3:** Comparison of objective knowledge between fellowship interest and no fellowship interest

Objective Knowledge Percentile Score	FI +	FI -	Unadjusted P-value+	Adjusted P-value*
			
	Mean (95% CI)*	Mean (95% CI)*		
Overall Score	56.44 (37.26, 75.61)	31.67 (5.27, 58.07)	<0.01	0.04

General Internal Medicine	48.47 (27.45, 69.50)	34.63 (5.68, 63.58)	0.09	0.27

Cardiology	58.09 (39.14, 77.05)	35.40 (9.30, 61.50)	0.02	0.05

Nephrology	52.04 (33.76, 70.33)	26.33 (1.16, 51.51)	0.01	0.02

Pulmonology	41.39 (22.18, 60.59)	20.09 (-6.36, 46.53)	0.03	0.07

Gastroenterology	45.22 (23.93, 66.50)	43.65 (14.35, 72.95)	0.62	0.90

Rheumatology	47.92 (28.30, 67.54)	35.62 (8.61, 62.63)	0.12	0.29

Inf. Diseases	44.72 (28.76, 60.68)	19.61 (-2.36, 41.58)	0.01	0.01

Hem/Onc	59.09 (39.13, 79.04)	28.38 (0.91, 55.85)	<0.01	0.01

+ Two-sample T-test; (FI +) Fellowship Interest; (FI -) No Fellowship Interest

* Analysis of Covariance (ANCOVA) adjusting for CMS, years since graduating, and previous training; Means are from adjusted analysis; PGY excluded from adjusted model because of correlation with fellowship interest

In a series of ANOVAs, statistically significant and plausible confounding demographic variables for the relationship between fellowship interest and objective general medical knowledge included years since graduation (p = 0.02), country of medical school (CMS; p = 0.06) and previous clinical training (p = 0.04). International Medical Graduates (IMGs) had higher scores than graduates of US medical schools while residents with prior formal clinical training and higher number of elapsed years since graduating from medical school had higher scores than their counterparts without prior training and with lesser time-lengths since graduating. Additional analysis using logistic regression to model fellowship interest with demographic variables (Table 1) returned post-graduate year of training as the only significant variable (p = 0.03).

After adjusting for country of medical school, years since graduation and previous training, the observed relationship between fellowship interest and objective general medical knowledge was preserved across all content areas. The statistical significance of the relationship between fellowship interest and overall medical knowledge was also preserved albeit lessened by the adjusted variables (p = 0.04). The adjusted mean difference in content-area specific percentile scores was significant only for nephrology, infectious diseases and hematology/oncology. (Table 3)

The need for general knowledge and the need for examination preparedness did not differ significantly as factors influencing study patterns between residents with and without fellowship interest. However, residents with fellowship interest were more likely to study so as to be competitive, compared to residents without fellowship interest (p = 0.03). (Table 4)

**Table 4:** Comparison of factors influencing study pattern between fellowship interest and no fellowship interest

	FI+			FI-			
		
	None	Some	Large	None	Some	Large	
		
Factor	N (%)	N (%)	N (%)	N (%)	N (%)	N (%)	P-value*
Need to be competitive	3 (9)	18 (51)	14 (40)	3 (38)	5 (62)	0 (0)	0.03

Need for general knowledge	0 (0)	6 (17)	29 (83)	1 (12)	3 (38)	4 (50)	0.06

Need for exam preparedness	5 (14)	15 (43)	15 (43)	2 (25)	5 (63)	1 (12)	0.33

* Pearson Exact Chi-square test

## Discussion

This study found that a significant percentage of internal medicine residents were interested in pursuing sub-specialty fellowship. A typical internal medicine resident with fellowship interest at the institution surveyed was more likely to be male, international medical graduate, aged 29 or less with no prior formal clinical training prior to residency and with interests in Cardiology, Pulmonology or Hematology/Oncology.

The authors hypothesized that internal medicine residents would devote much study and research time to their subspecialty of interest in order to remain competitive for fellowship applications. This observation is supported by two findings in this study. Firstly, the finding that the need to be competitive was a major and significant factor that influenced the study patterns of IM residents with fellowship interest compared to their counterparts without fellowship interest; Secondly, the finding that a majority (75%) of IM residents with fellowship interest had spent at least 2-3 months rotating through their sub-specialty interest areas.

Data relating to the associations between fellowship interest and medical knowledge of internal medicine residents is lacking. Our working hypothesis prior to the conduct of this study was that based on the need to be competitive in their chosen fields, leading to proportionately less study of general internal medicine concepts, residents with fellowship interest would have lesser knowledge of general internal medicine.

A number of factors could potentially confound this hypothesis, including 1) Time-length since graduating from medical school and 2) Training in internal medicine (or other specialty) prior to current residency. A resident with fellowship interest could have lower IM-ITE scores, not because of his interest in fellowship training but because he had lost touch with academic medicine due to a long interval between graduation and commencement of residency training. Likewise, a resident without fellowship interest could have higher scores due to knowledge acquired from prior training in internal medicine.

Even though there was no significant difference in the groups with respect to these potential confounders, in multivariable analysis, they (in addition to gender and CMS) significantly confounded the relationship between fellowship interest and objective general medical knowledge. From our analysis of potential confounders, males had higher scores than females; International Medical Graduates (IMGs) had higher scores than graduates of US medical schools. Also residents with prior formal clinical training and higher number of elapsed years since graduating from medical school had higher scores than their counterparts without prior training and with lesser time-lengths since graduating. While the confounding effects of prior clinical training and number of years since graduation have real world plausibility, the confounding effect of gender is not easily explicable. With regards to Country of Medical School, IMGs are known to outperform U.S. graduates from assessments of IM-ITE scores up to the year 2000 [17]. IM-ITE data from more recent years however is yet unpublished.

None of the confounding demographic variables had a direct relationship with the primary independent variable (fellowship interest). After adjusting for plausible confounders, Internal Medicine residents with fellowship interest had statistically significantly higher overall percentile scores.

The intriguing finding of greater scores among residents with fellowship interest, even after multivariable adjustment, does not support our original hypothesis. Are residents with fellowship interest more knowledgeable than their counterparts at baseline? Or does the competitive drive to excel make them better as they progress through residency?

Our study is not without limitations. The interpretation and generalizability of our results is hindered by sample size and other limitations. Among the survey respondents, eight (8) had no interest in pursuing subspecialty fellowship training and quite a few subgroups derived from this control group include only one or two persons. This is especially important as it relates to certain confounding variables that were adjusted for. Our sample size may not have allowed for an effective evaluation of the differential effects of the identified confounding factors on the observed results. Our study was also limited to one cohort of each residential year of training in a single institution in which only 13% of trainees were graduates of US medical schools.

In trying to study factors that affect a resident's knowledge within a limited time frame defined by a 3-yr residency training format, further limited by our independent variable only being definable in the second year of residency training, our study was designed as a cross-sectional study. This is not without its own limitations. If possible, a prospective study with repetitive measurements of objective examination knowledge would provide more valid results. This is important as it is possible that residents who have less general knowledge and are not planning on a fellowship do not yet do so because they still lack the necessary prerequisite general knowledge

In view of these limitations, the demonstrated associations should not be interpreted in a causal context as having established a causative relationship between career intentions and level of knowledge (or vice versa). Despite the single study site and sample size limitations, the consistency of the findings and the lack of statistically significant differences in the groups with respect to factors that may have biased general medical knowledge suggest that this early finding may be indicative of a more general phenomenon in IM training.

There is an ongoing debate over whether IM residency education needs to be redesigned to respond to changes in medical practice and health care delivery. Some recommend that IM training be lengthened to 4 years so that residents can fully grasp the depth and width of current knowledge. Others suggest shortening the training to 2 years so that residents can quickly advance to subspecialty fellowship training. The debate needs to be better informed by empirical data, and our study represents an initial attempt to provide such data.

## Conclusions

We studied the knowledge performance of residents with and without aspirations to pursue subspecialty training, and found those interested in fellowship training to perform better on the IM-ITE in terms of overall medical knowledge. In our ongoing work, we are continuing data collection among residents in both university-affiliated and community programs to determine whether residents who have a specialty interest demonstrate greater knowledge in their own chosen specialty compared to other specialties.

## Competing interests

The authors declare that they have no competing interests.

## Authors' contributions

URO conceived of and designed the study and was involved with data acquisition, analysis and interpretation as well as article drafting and revision. EBL carried out data analysis and revision of draft article. PH made substantial contributions to data analysis and interpretation and revised the article for important intellectual content. ACY made substantial contributions to study design and data acquisition and revised the article for content. All authors read and approved the final manuscript.

## Pre-publication history

The pre-publication history for this paper can be accessed here:

http://www.biomedcentral.com/1472-6920/11/5/prepub

## Supplementary Material

Additional file 1 **Survey Instrument**. Click here for file
