# Supplementary material for: Associations between subspecialty fellowship interest and knowledge of internal medicine: A hypothesis-generating study of internal medicine residents
Source: BMC Med Educ. 2011 Jan 31;11:5. doi: 10.1186/1472-6920-11-5 (PMC3038163; doi:10.1186/1472-6920-11-5)
Supplement: Additional file 1 — Survey Instrument. [file 1472-6920-11-5-S1.PDF]

Pilot Residency Knowledge Survey

1. INTRODUCTION

Thank you for taking out time to complete this short survey. We really appreciate it. We are trying to determine if a resident's general medical knowledge is in any way affected by a decision to do or not to do a fellowship.Your input will help us answer this question and also help us understand better, factors that affect resident study patterns and fellowship choices.
The answers you provide will be completely non-identifying and will remain strictly confidential.

Uchenna Ofoma
Jana Preis
Andrew Yacht

2. BACKGROUND INFO

Part 1 of this survey requires you to provide us with some background information about you and your medical training

* 1. How old are you?

- 29 or less
- 30-34
- 35-39
- 40 or older

* 2. Indicate your sex

- Male
- Female

* 3. In what year of Internal Medicine training are you currently in?

- PGY1
- PGY2
- PGY3

* 4. Please indicate country of medical school

* 5. Please indicate approximate date of graduation from medical school. (MM/DD/YYYY)

* 6. Have you had any previous formal clinical training after leaving medical school and prior to Internal Medicine Residency in the United States? Please include previous residencies or fellowships. PLEASE DO NOT INCLUDE OBSERVERSHIPS OR EXTERNSHIPS!

- Yes
- No

Pilot Residency Knowledge Survey

3. PRIOR FORMAL TRAINING

Please tell us a little bit more about your previous formal clinical training prior to internal medicine residency in the United States

* 1. Please indicate your specialty of formal clinical training after leaving medical school and prior to Internal Medicine residency in the United States. No abbreviations please!

* 2. Please indicate the length of your previous formal clinical training prior to Internal Medicince Residency

- 0-1yrs
- 2-3yrs
- 4 and above

4. FELLOWSHIP CHOICES (I)

In this section, you will be required to answer 4 question pertaining to fellowship choices.

* 1. Which of the following statements best applies to you?

- I have already secured a fellowship position
- I am currently involved in the fellowship application process
- I was not successful in my fellowship application
- I have not participated in the fellowship application process, but plan to do so in the future
- I have not participated in the fellowship application process and do not intend to do so

5. FELLOWHIP CHOICES (II)

Completing this page indicates that you are in some way interested in doing a fellowship. This means that you are in the process of, or previously participated in the fellowship application process, or you intend to participate in the fellowship application process in the future. If you are not interested in doing a fellowship, please return to the previous page and select the last option.

* 1. To what extent did each of the following factors influence your choice of fellowship?

	No extent	Some extent	Large extent
Prestige	◯	◯	◯
Career Advancement	◯	◯	◯
Personal Satisfaction	◯	◯	◯
Financial Rewards	◯	◯	◯
Other (please specify below)	◯	◯	◯

Please indicate 'Other' factor in the box

* 2. In what year of your residency did you commit yourself to your fellowship choice?

- ◯ Prior to commencing residency
- ◯ PGY1
- ◯ PGY2
- ◯ PGY3

* 3. Excluding the general medical floors, how much rotation experience (MICU, CCU, elective, etc.)did you obtain in the medical field of your fellowship choice?

- ◯ 0 month
- ◯ Less than 1 month
- ◯ 1 month
- ◯ 2-3 months
- ◯ 4 and more

Pilot Residency Knowledge Survey

* 4. Please indicate your choice of fellowship subspecialty. (It does not matter if you have not applied for a fellowship yet or if you were not successful in your fellowship application) If you will be doing a research fellowship, please indicate in the space provided and include the specialty of the research fellowship.

- Cardiology
- Hematology and Oncology
- Infectious diseases
- Nephrology
- Endocrinology
- Gastroenterology
- Pulmonary Medicine
- Rheumatology
- Pulmonary Medicine with Critical Care
- Critical Care with other specialty
- General Internal Medicine

Other (please specify)

Pilot Residency Knowledge Survey

6. CHOICES OTHER THAN FELLOWSHIP

Completing this page indicates that you have not participated in and will not be participating in the fellowship application process. If this does not apply to you, please return to the previous page and choose the option that best describes your fellowship interest.

* 1. How would you best describe your immediate plans at the completion of Internal Medicine residency?

- Primary Care (outpatient) Medicine
- Hospitalist (inpatient) Medicine
- A combination of Inpatient and Outpatient Medicine
- Research
- Residency in another medical field

7. KNOWLEDGE/STUDY PATTERNS

In this section, please rate as accurately as possible your subjective medical knowledge of the various medical subspecialties.

* 1. Please rate your knowledge of the various medical subspecialties

	Poor	Fair	Good	Excellent
General Internal Medicine including Primary Care & Hospitalist Medicine				
Cardiology				
Hematology and Oncology				
Infectious Disease medicine				
Nephrology				
Endocrinology				
Gastroenterology				
Neurology				
Pulmonary Medicine				
Rheumatology				
Critical Care Medicine				

* 2. There are 11 medical subspecialties listed below. A resident who divides study time equally among them would spend approximately 9% of his/her time studying each. Using your own study experience, please indicate the relative amount of time you generally spend studying each medical subspecialty.
As much as possible, please ensure that the total of all the choices you select approximates 100%

	0-10%	11-20%	21-30%	31-40%	41-50%	51-60%	61-70%	71-80%	81-90%	91-100%
General Internal Medicine including Primary Care & Hospitalist Medicine										
Cardiology										
Hematology and Oncology										
Infectious Disease Medicine										
Nephrology										
Endocrinology										
Gastroenterology										
Neurology										
Pulmonary Medicine										
Rheumatology										
Critical Care Medicine										

Pilot Residency Knowledge Survey

* 3. There are three factors listed below. To what extent did each of the factors influence your study pattern

	No extent	Some extent	Large extent
the need to be competitive in the medical field of your fellowship choice			
the need to have a general knowledge of medicine			
the need to be well prepared for the board exams			

8. PRACTICE PLANS

This is the final part of this survey. Please tell us a little bit about your future practice plans by answering the two questions outlined below.

*** 1. Which of the following best describes your plans for medical practice at the completion of your residency and/or fellowship training?**

- I plan to practice general internal medicine (primary care and/or hospitalist medicine)
- I plan to practice both general medicine and the subspecialty of my fellowship training
- I only plan to practice in the area of my subspecialty training
- I have no immediate plans for medical practice
- I plan to go into medical research

*** 2. Irrespective of your future plans,which of the following do you think will best describe your level of comfort with patient care after completing your Internal Medicine residency training?**

- I will be comfortable caring for all patients irrespective of the scope of their ailments
- I would prefer not to be directly responsible for patients presenting with ailments outside the scope of my subspecialty
- I would base my decision to be directly responsible for patients presenting with ailments outside the scope of my subspecialty on the nature and/or severity of the ailment

Pilot Residency Knowledge Survey

9. THANK YOU

Thank you for taking out time to complete the survey. Please click "Done" below to submit your answers.

Uchenna Ofoma
Jana Preis
Andrew Yacht
